# A primary mechanism for efficacy of the ketogenic diet may be energy repletion at the tripartite synapse

**DOI:** 10.1088/1741-2552/adef7f

**Published:** 2025-07-24

**Authors:** Shubhada N Joshi, Aditya N Joshi, Narendra D Joshi

**Affiliations:** 1 National Center for Adaptive Neurotechnologies (NCAN), Stratton VA Medical Center, Research 151, 113 Holland Ave., Albany, NY 12208, United States of America; 2 University of Pennsylvania Perelman School of Medicine, Philadelphia, PA, United States of America; 3 Retired, Niskayuna, NY, United States of America

**Keywords:** ATP, cell metabolism, energy metabolism, ketone, ketogenic diet, epilepsy

## Abstract

*Objective.* The ketogenic diet is a well-known treatment for epilepsy. Despite decades of research, it is not yet known how the diet accomplishes its anti-seizure efficacy. One of the earliest proposed mechanisms was that the ketogenic diet is able to replenish cellular energy stores in the brain. Although several mechanisms have been suggested for how energy depletion may contribute to seizure generation and epileptogenesis, how the dynamics of energy depletion actually leads to abnormal electrical activity is not known. *Approach.* In this work, we investigated the behavior of the tripartite synapse using a recently developed neurochemical model, which was modified to include ketone chemistry. We ran transient, non-steady-state simulations mimicking normoglycemia and ketosis for metabolic conditions known to be clinically treated with the ketogenic diet, as well as a condition for which the ketogenic diet was not effective clinically. *Main results.* We found that reduction in glucose, as well as pathological decreases in the activity of glucose transporter 1, pyruvate dehydrogenase complex, monocarboxylate transporter 1 (MCT1), and mitochondrial complex I, all led to functioning of the tripartite synapse in a rapid burst-firing mode suggestive of epileptiform activity. This was rescued by the addition of the ketone D-*β*-hydroxybutyrate in the glucose deficit, glucose transporter 1 deficiency, and pyruvate dehydrogenase complex deficiency, but not in MCT1 deficiency or mitochondrial complex I deficiency. *Significance.* We demonstrated that replenishment of cellular energy stores is a feasible mechanism for the efficacy of the ketogenic diet. Although we do not rule out other proposed mechanisms, our work suggests that cellular energy repletion may be the primary action of the ketogenic diet. Further study of the contribution of energy deficits to seizure onset and even epileptogenesis may yield novel therapies for epilepsy in the future.

## 1. Introduction

It is widely accepted that the primary fuel used by the brain, at least as a whole, is glucose [1–4]. It has been reported that in humans, the brain consumes about 20% of the total O_2_ used by the body at rest, even though it only comprises approximately 2% of the body mass [5]. This figure is only an estimate of the percentage of energy used by the brain, as it does not necessarily capture the fraction of energy consumed by anaerobic metabolism. This suggests that on a weight basis, the brain requires much more energy than most other organs in the body. What does the brain do, then, if glucose supplies are scarce? During prolonged fasting, it has been shown that glucose synthesis from fat and protein catabolism is insufficient to support the glucose requirements of the brain [6].

In conditions of low glucose availability, the body has evolved another mechanism to produce energy: the synthesis and then oxidation of ‘ketone bodies’. Ketone bodies include D-*β*-hydroxybutyrate (henceforth called *β*-hydroxybutyrate), acetoacetate, and acetone; the former two are synthesized by hepatocytes, while acetone forms from spontaneous decarboxylation of acetoacetate. The liver synthesizes ketone bodies when there is low glucose availability, which may result from low dietary availability (starvation, prolonged fasting, or low-carbohydrate diet), or inability to use available glucose (too low insulin or high insulin resistance) [7]. In such low carbohydrate conditions, ketones become the primary energy source for the brain, and in fact, it has been shown that ketones can supply up to approximately 60% of the brain’s metabolic energy requirements [6, 8]. Thus, ketones may be an important class of substrate that maintains energy balance in the brain. Although the body typically synthesizes ketones in the condition of pathologic low availability of glucose, so-called ‘ketogenic diets’, which are *intended* to generate ketones for energy use without causing nutrient deprivation, have been investigated as a potential therapeutic intervention. These diets have long been known to effectively treat the neurological disease of epilepsy [9–11].

Numerous mechanisms have been advanced for the efficacy of the ketogenic diet in epilepsy. These include possibilities as diverse as effects at the membrane permeability transition pore of mitochondria, alteration of the balance between pro- and anti-apoptotic factors through effects on Bcl-2-associated agonist of cell death, altered utilization of glutamate (the primary excitatory neurotransmitter in the brain) and/or *γ*-amino-butyric acid (GABA, the main inhibitory neurotransmitter in the brain), altered loading of synaptic vesicles, effects on the ATP-sensitive potassium (*K*_ATP_) channel, modulation of gene expression through histone deacetylases, and reduction of inflammation, among others [11–17]. A very early theory to explain how the ketogenic diet is beneficial for the treatment of seizures centered around the idea that the ketogenic diet increases cellular energy stores, as evidenced by increased ATP/ADP ratio [18]. Other groups have found that instituting a ketogenic diet leads to increased mitochondrial metabolism and flux through the tricarboxylic acid cycle, increased mitochondrial biogenesis, and increased astrocytic anaplerotic metabolism to synthesize oxaloacetate for use in the tricarboxylic acid cycle, supporting this hypothesis [13, 19, 20].

We are not aware of any models that have assessed the potential benefits of a ketogenic diet analytically. Indeed, most of the hypothesized effects of the ketogenic diet cannot be modeled by electrical-only models such as integrate-and-fire models or models based on the Hodgkin–Huxley formalism. These models rely on changes in various currents to demonstrate changes in excitability, and several groups have connected metabolic processes to currents (such as [21, 22]). However, electrical-only models cannot model purely metabolic or non-signaling effects, such as alteration in synaptic vesicle loading or changes in protein activity caused by modulation of gene expression. We recently developed a novel ‘neurochemical’ model of an excitatory tripartite synapse (comprised of a neuronal presynaptic terminal, neuronal post-synaptic terminal, an astrocytic process, synaptic cleft, and extracellular fluid) and its associated vasculature within a network, which included various intracellular biochemical processes, electrical phenomena, and network inputs [23]. Because this model includes transport processes, enzyme activities, and vesicular processes, in addition to electrical processes, a significantly broader range of metabolic interventions can be simulated to investigate possible mechanisms of the ketogenic diet, compared to the electrical-only models.

We hypothesize that ameliorating energy deficits is a major mechanism of efficacy of the ketogenic diet for the treatment of seizures. Specifically, we theorize that provision of ketones will increase the amount of substrate available for energy generation in tripartite synapse systems, allowing them to come out of the pathological state of rapid burst firing that is the presumed correlate of epileptiform activity and seizures. In this work, we modify our prior ‘neurochemical’ mathematical model to include ketone chemistry, and we demonstrate that elevated levels of ketone bodies can rescue energy deficiency at the tripartite synapse. Provision of ketones is capable of normalizing function at the tripartite synapse in disease states where metabolism of these substrates bypasses the abnormality, but is not able to rescue downstream deficiencies. The efficacy of the ketogenic diet in the treatment of seizures suggests that energy deficiency plays a significant role in their generation.

## 2. Methods

### 2.1. Model specification

The model was developed in the SimBio environment of MATLAB R2018b (Natick, MA), as a system of ordinary differential equations representing 159 reactions (reactions, compartments, parameters, initial values of species, events, and rules are in the supplementary materials). The model was run on a Dell 5820 workstation (Dell Computers, Round Rock, TX). The equations were solved using the ode15s solver in MATLAB, with a maximum step size of 2 ms.

**Figure 1.**
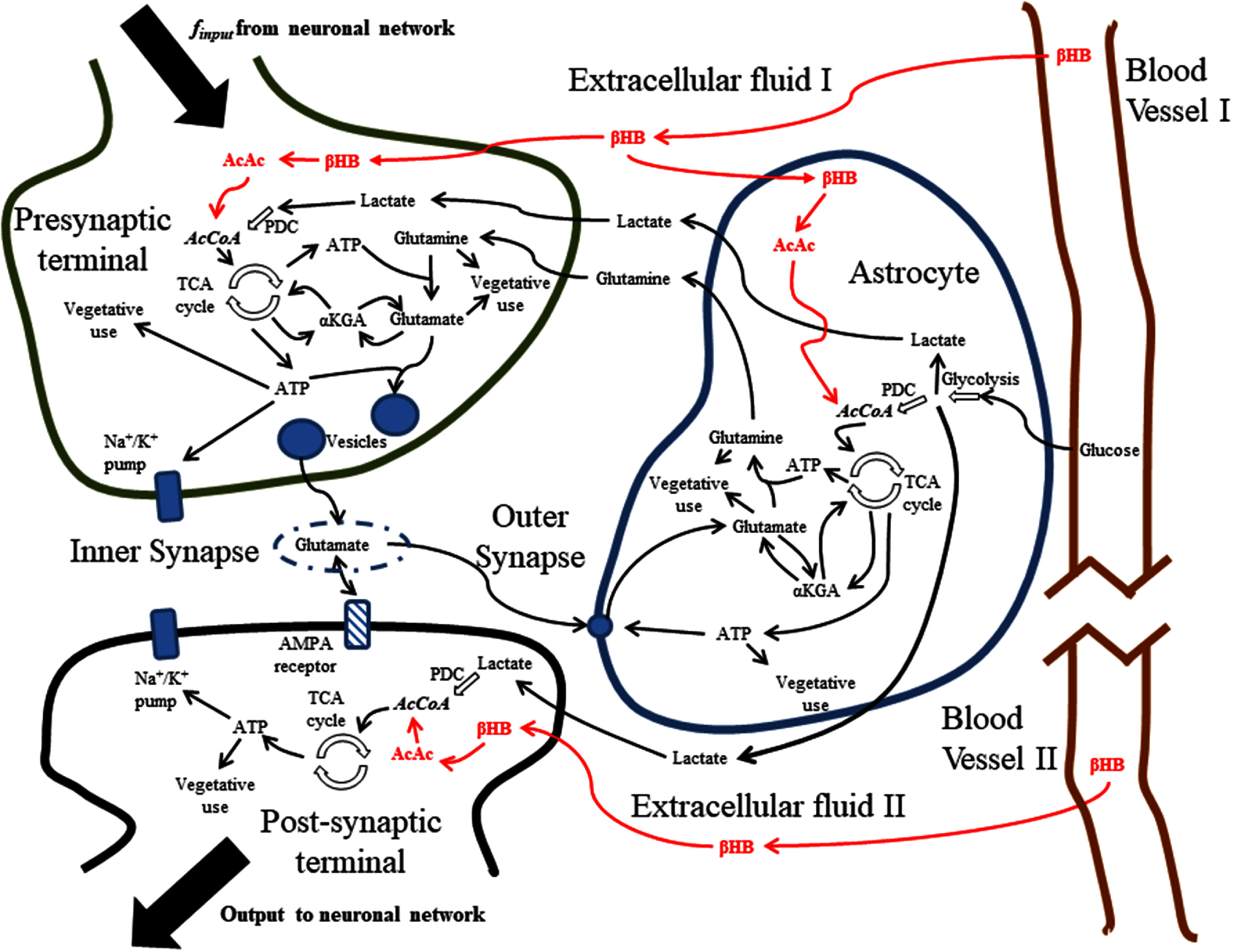
Schematic of the tripartite synapse with ketone reactions. The model is a transient (time-accurate, non-steady-state) analytic model of the tripartite synapse within a network, including key processes involved in energy generation and glutamatergic neurotransmission. The figure is adapted from [23], CC BY 4.0, to show the ketone reactions (in bold and red). AcetylCoA has been explicitly shown (italicized) to illustrate where the ketone and glucose/lactate pathways converge. PDC = pyruvate dehydrogenase complex. TCA cycle = tricarboxylic acid cycle. *α*KGA = *α*-ketoglutarate. *β*HB = *β*-hydroxybutyrate. AcAc = acetoacetate. AcCoA = acetyl-coenzyme A.

The underlying model was a recently developed multi-compartment ‘neurochemical’ model, which comprised the presynaptic terminal, post-synaptic terminal, and astrocytic component of an excitatory tripartite synapse, as well as the blood vessel, synaptic cleft, and extracellular fluid compartments. Generation and reuptake of neurotransmitter, loading into synaptic vesicles, transient binding at the post-synaptic terminal for signaling, and ion movements leading to changes in membrane potential, constituted the neurotransmitter processes. The major chemical reactions involved in the generation and utilization of ATP were modeled to capture synaptic energetics [23]. Modifications to the model to include ketone chemistry are described below. The primary processes modeled are depicted in figure 1.

Network processes were modeled using decision logic, which determined the input firing frequency from the network, and whether or not there was a ‘firing’ event (release of a synaptic vesicle) in the presynaptic terminal. Several ‘critical’ parameters determined the network input into the tripartite synapse system. The variable component of the input firing frequency (*ν*) represented the sum effect of network input into the tripartite synapse after summation at the axon hillock. This was represented as a single firing frequency of action potentials down the axon, which is upstream of the presynaptic terminal in the model. The extinction parameter (*ζ*) was an abstract parameter that determined the conditions under which the network would drop from a state of high rate of firing into a quiescent state (an extinction phenomenon). The constant component of the ‘network’ input firing frequency (*ϵ*) represented the rate of spontaneous firing of the presynaptic terminal, which is an intrinsic property of the tripartite synapse. In the normal mode of operation, *ν* took on a predetermined non-zero value, and *ν*+ *ϵ* represented the effective input firing frequency from the network. If the tripartite synapse system met conditions for extinction (as determined by *ζ*), then *ν* dropped to 0, indicating that the entire network had dropped into a quiescent state, and there was no network input. The only synaptic vesicle release occurred due to the spontaneous rate of firing (*ϵ*) of the presynaptic terminal. When the tripartite synapse system recovered sufficient resources (neurotransmitter and ATP), it ‘reignited’, and *ν* returned to a non-zero value, indicating that the network was significantly contributing to the input again. All of these parameters appear only in the decision logic of the model, and how variation in these parameters affected the model is described elsewhere [23]. **The critical parameters *ν, ϵ*, and *ζ* were NOT varied for any of the simulations in this work.**

### 2.2. Modifications to the model to include ketone chemistry

In order to include ketone chemistry, transport processes were added for the entrance of *β*-hydroxybutyrate from the blood vessel compartments into the extracellular fluid compartments, and then for the uptake of *β*-hydroxybutyrate into each of the cellular compartments. Each cellular compartment had reactions for the sequential conversion of *β*-hydroxybutyrate into acetoacetate, acetoacetylCoA, and acetylCoA. Because no intracellular compartments were included in the model, the cytosolic and mitochondrial reactions for the conversion of acetoacetate to acetoacetylCoA were included in parallel. The cytosolic and mitochondrial thiolase reactions (which interconvert acetoacetylCoA and two acetylCoA) were combined because the reactants and products are the same [24, 25]. The differential equations for certain species (NAD, NADH, succinylCoA, succinate, acetylCoA, ATP, and ADP) in each compartment were modified to account for interactions with the ketone chemistry. AMP was added as an additional species.

For simplicity, and because the concentrations of acetoacetate and acetone are significantly lower than that of *β*-hydroxybutyrate, source terms for the former two species were not included in the blood vessel compartments [7]. Concentrations of glucose and *β*-hydroxybutyrate in the simulations for ‘normal’ diet and ketogenic diet were taken in the range of published values [26–30]. Of note, the glucose concentrations utilized were at *in vivo* levels, whereas the prior model used glucose levels more consistent with those required to maintain *in vitro* slice cultures [23, 31]. In order to maintain the model’s behavior with the lower glucose concentrations *in vivo*, the glucose transporter reaction rate constant *k*_f_ was proportionally increased to maintain the same glucose flux into the astrocyte. In addition, several small adjustments were made to other parameters to make the transitions similar to those of the prior model (please see ‘parameters’ in supplementary materials). The availability of coenzyme A was assumed to be not limiting. It was assumed that transport of lactate would not competitively inhibit transport of *β*-hydroxybutyrate and vice-versa through the monocarboxylate transporter 1 (MCT1).

### 2.3. Terminology and summary of regimes

In our prior work, we found that the relative balance between availability of glutamate for neurotransmission, and ATP for neurotransmission and other processes, led to different regimes of operation of the tripartite synapse system [23]. When the firing of the tripartite synapse system was purely limited by availability of glutamate for neurotransmission (represented by availability of synaptic vesicles filled with glutamate), the presynaptic membrane potential showed a pattern of alternating UP states (periods of rapid firing with relatively depolarized membrane potential) and DOWN states (periods of quiescence with relatively hyperpolarized membrane potential). In this state, termed ‘Regime C’, the presynaptic terminal was able to fire at the same rate as the input frequency from the network for the entire duration of the UP state. If the primary limitation was due to availability of glutamate for neurotransmission, but there was superimposed limitation of ATP, the tripartite synapse system still showed alternating UP and DOWN states, but was only able to fire at the same rate as the network input frequency for the early part of each UP state. In this mode, termed ‘Regime D’, the superimposed ATP limitation manifested as the presynaptic terminal firing at a lower frequency than the network input frequency for the latter part of each UP state. UP and DOWN states are present in physiological systems, and are a normal firing pattern. However, when the tripartite synapse system transitioned into a mode where availability of energy (ATP) was the primary limiting resource, it showed a very different firing pattern: very brief bursts of rapid firing (UP states) with equally brief bursts of quiescence (DOWN states). These states were approximately one order of magnitude shorter than the corresponding states in Regime D. This mode was termed ‘Regime E’, and its presence demonstrated that the firing behavior of the tripartite synapse system was materially altered by the availability of energy. It was speculated that the pattern of rapid bursts of firing and quiescence could represent the synaptic-level equivalent of epileptiform electrical activity, thus establishing resource limitation as a possible mechanism for the generation of this activity [23]. These findings are summarized in table 1 below; Regime D and Regime E are referred to throughout the work. In addition, *k*_f_ refers to forward reaction rate constant, and *k*_r_ refers to reverse reaction rate constant throughout the work.

**Table 1.** Summary of regimes of interest in the model.

Regime	Limited by	Duration of UP and DOWN states	Interpretation
Regime C	Neurotransmitter	Long	**Normal physiological.** Presynaptic terminal always fires at the same rate as the input during the UP state.
Regime D	Neurotransmitter, with superimposed energy limitation	Long	**Normal physiological.** Presynaptic terminal initially fires at the same rate as the input during the UP state. When energy limitation sets in, the presynaptic terminal firing rate drops, but the network does not extinguish. The network extinguishes when the presynaptic terminal runs out of vesicles.
Regime E	Energy	Short	**Pathological.** Presynaptic terminal is only briefly able to fire at the same rate as the input during the UP state, prior to energy deficit. **This state may be the single-synaptic correlate to seizure-like activity.**

### 2.4. Simulations

We chose a *ν* of 10 Hz, *ϵ* of 0.3 Hz, and *ζ* of 0.5, which demonstrated UP and DOWN states in Regime D, for all of the simulations, in order to demonstrate potential transitions into Regime E. The values of these three critical parameters were kept constant for all of the simulation runs. In addition, all of the initial conditions and parameters (found in the supplementary materials) were kept constant except for the variable(s) of interest in each experiment. It had previously been shown that the duration of the UP and DOWN states were significantly different between Regime D and Regime E. Regime C was distinguished from Regime D by observing that the moving average output frequency of the presynaptic terminal was equal to the input frequency (*ν*+ *ϵ*) for the entire UP state duration in Regime C, whereas it was lower than the input frequency for part of the UP state in Regime D [23]. Which regime any particular simulation run belonged to was determined by the identity of the final complete cycle (UP and DOWN state together) at the end of the simulation. No simulated runs were performed in Regime C because it has been suggested that at least some neurons function near the top of their metabolic capacity [32]. The identification of regime in a simulated run was determined by the duration of the final complete cycle (long—Regime D; short—Regime E).

To determine a minimum ATP generation rate that can distinguish between Regime D and Regime E, we ran simulations varying the concentration of glucose in the blood vessel, with two different concentrations of *β*-hydroxybutyrate (‘normal’: 0.25 mM, and ‘ketogenic’: 2 mM). The concentrations of *β*-hydroxybutyrate were kept identical in the first and second blood vessel compartments. The average rate of ATP generation by complex V of the electron transport chain, and by succinylCoA synthetase (the only other active reaction that generates ATP) in the presynaptic terminal were calculated during the simulation. This was accomplished by calculating the generation rate of ATP (in units of concentration/time) at each time point of the simulation: *k*_f, complex V_ * [energy token] * [ADP] for complex V, and *k*_f, succinylCoA synthetase_ * [succinylCoA] * [ADP] for succinylCoA synthetase. Note that [energy token] represents the concentration of an energy equivalent (created by transportation of a hydrogen ion across the mitochondrial membrane during the redox reactions of the electron transport chain) that will ultimately lead to generation of ATP by complex V [23]. These generation rates were averaged over the last 200 s of the simulation to exclude the starting transient. They were converted to units of molecules ATP/time by multiplying by the volume of the presynaptic terminal and Avogadro’s number.

We also ran simulations varying both the concentration of glucose and the concentration of *β*-hydroxybutyrate to explore the bifurcation between Regime D and Regime E as a function of the concentrations of these two species. The concentrations of *β*-hydroxybutyrate were kept identical in the first and second blood vessel compartments. To investigate whether addition of *β*-hydroxybutyrate could rescue energy deficit caused by low glucose, we simulated conditions of normal concentrations of glucose and ketone, low glucose with normal ketone, and low glucose with elevated ketone (representing concentrations obtained with a ketogenic diet). The ‘normal’ glucose level was set at 5 mM (90 mg dl^−1^), and the ‘normal’ level of *β*-hydroxybutyrate was set at 0.25 mM [26–30]. ‘Low’ glucose was set at 3.5 mM (≈ 63 mg dl^−1^) and ‘elevated’ ketone was set at 2 mM. We also characterized the bifurcation between Regime D and Regime E further by plotting phase plane diagrams of ATP versus number of vesicles filled with glutamate for Regime D (glucose 5 mM, *β*-hydroxybutyrate 0.25 mM) and Regime E (glucose 3.5 mM, *β*-hydroxybutyrate 0.25 mM).

We next ran simulations of various pathological conditions to investigate whether increased levels of ketone could rescue pathological firing patterns. In each case, we decreased the relevant reaction rate constant(s) until we observed that there was a state transition from Regime D to Regime E, under ‘normal’ conditions of glucose (5 mM) and *β*-hydroxybutyrate (0.25 mM). We then decreased glucose to 3.5 mM to represent the level of glucose seen in the ketogenic diet, and increased the concentration of *β*-hydroxybutyrate until the firing pattern transitioned back from Regime E into Regime D. If we had to escalate the concentration of *β*-hydroxybutyrate to 10 mM and the system still did not transition back into Regime D, then we held that the conditions of the ketogenic diet were unable to rescue the pathological condition. For glucose transporter 1 deficiency, we decreased the forward reaction rate constant for the glucose transporter on the astrocytic process. For pyruvate dehydrogenase complex deficiency, we decreased the rates of all three reactions of the pyruvate dehydrogenase complex (please see ‘reactions’ in supplementary materials) in each compartment (presynaptic terminal, post-synaptic terminal, and astrocyte). The first reaction of the pyruvate dehydrogenase complex is non-competitively inhibited (see [23]); for this reaction, the maximum reaction rate constant *k*_0_ was decreased. For monocarboxylate 1 transporter deficiency, we decreased the rates of transport for lactate and *β*-hydroxybutyrate in the blood vessels and astrocyte (but NOT the presynaptic or post-synaptic terminals). For mitochondrial complex I, we decreased both rates of complex I activity (please see ‘reactions’ in supplementary materials) in the presynaptic terminal, post-synaptic terminal, and astrocyte.

### 2.5. Sensitivity analysis

**Figure 2.**
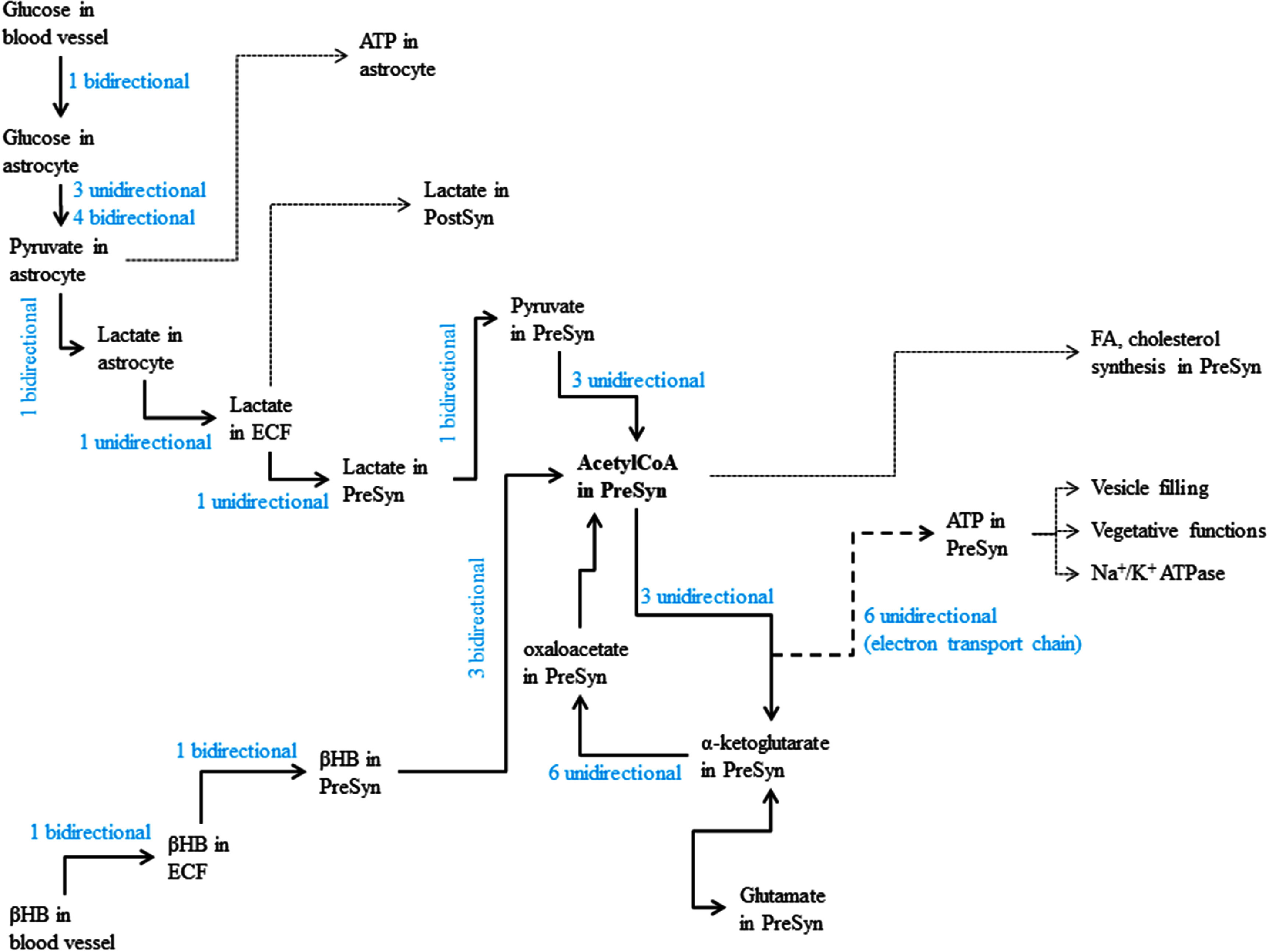
Energy pathway for sensitivity analysis. Major species and pathways connecting them are schematically represented for the sensitivity analysis. The text in blue indicates the number of reactions that were investigated for the sensitivity analysis. Bidirectional reactions are reversible, and thus sensitivity was analyzed for both the forward rate constant and the reverse rate constant (representing two separate reactions). Thin dashed lines denote downstream pathways that were not analyzed. The heavy dashed line represents the pathway for the electron transport chain, where ATP is not directly derived from the tricarboxylic acid cycle, rather it depends on reducing equivalents from the cycle. ECF = extracellular fluid. *β*HB = *β*-hydroxybutyrate. PreSyn = presynaptic terminal. PostSyn = post-synaptic terminal. FA = fatty acid.

In order to assess local robustness of the model, we performed a parameter-by-parameter sensitivity analysis to determine whether there were any specific parameters that significantly affected outcomes of the simulation runs. We identified the principal energy flow pathway for the presynaptic terminal (figure 2), starting at blood glucose and *β*-hydroxybutyrate, ultimately resulting in the generation of ATP in the presynaptic terminal. Each of the reaction rate constants was individually perturbed by ±10%, and simulations were performed at baseline conditions, ketogenic conditions, conditions of pathologic transition from Regime D to Regime E, and conditions of rescue with ketone resulting in a transition back from Regime E to Regime D. Of note, several of the reactions investigated were subject to allosteric modulation—either non-competitive inhibition or allosteric activation. In these cases, the minimum (for allosteric activation) or maximum (for non-competitive inhibition) reaction rate constant, denoted *k*_0_, was the parameter that was perturbed, rather than the final calculated reaction rate constant *k*_f_. For the first reaction of the pyruvate dehydrogenase complex reaction, and the first reaction of the *α*-ketoglutarate dehydrogenase complex, the *k*_0_ was shared for the presynaptic, post-synaptic, and astrocytic terminals. For pathologic conditions in which addition of ketone was unable to accomplish a transition from Regime E back to Regime D, simulations for sensitivity analysis were done with the highest tested level of ketone (10 mM *β*-hydroxybutyrate). The two most important species for the sensitivity analysis were glucose and *β*-hydroxybutyrate. We performed a full mapping of the effects of varying these two initial conditions elsewhere, and as such, perturbations of these species were not repeated in the sensitivity analysis.

We also performed a sensitivity analysis to explore the ranges of parameters that led to state transitions in the system. We investigated the same reactions identified above, and ran simulations just in the baseline condition, which exhibited Regime D (see below). We evaluated the changes in individual parameters required for the system to transition from Regime D into Regime E. Each parameter was iteratively perturbed until the transition region was identified to within ±1% or less. If there was no state transition with decreasing a forward reaction rate constant to 1% of baseline or increasing a reverse reaction rate constant to 100x of baseline, then it was held that the system was insensitive to changes in that reaction rate constant.

## 3. Results

### 3.1. There is a minimum ATP generation rate that sustains steady synaptic firing

**Figure 3.**
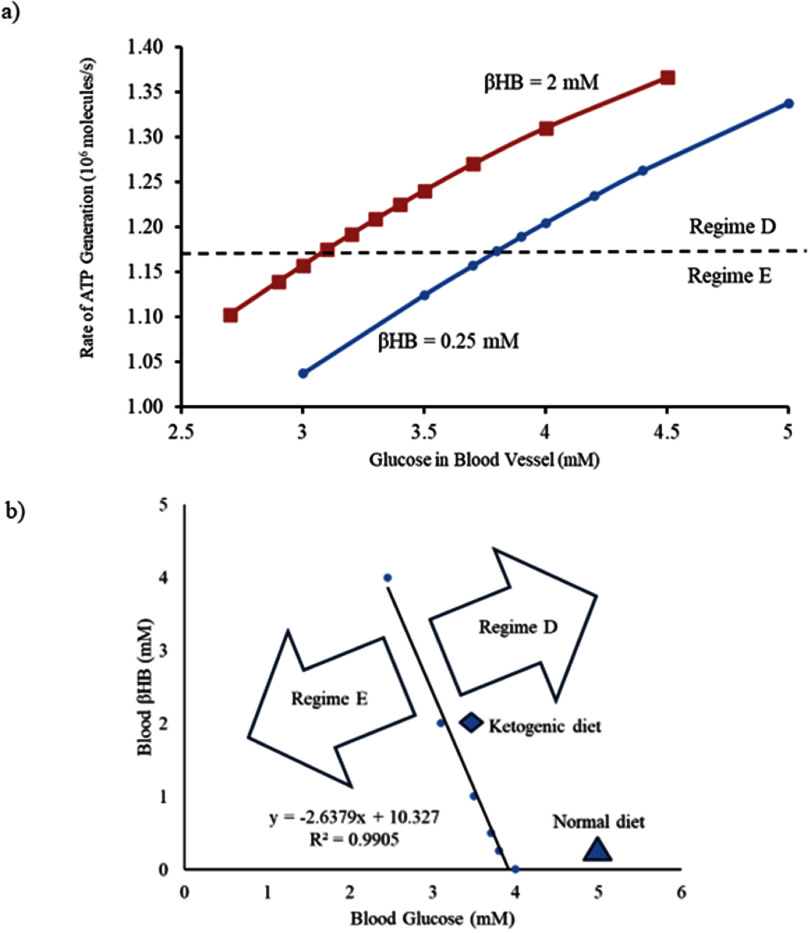
Rate of ATP generation in the presynaptic terminal of the tripartite synapse. (a) Average rate of ATP generation is calculated from 100 s to 300 s to exclude the starting transient (blue line: *β*-hydroxybutyrate 0.25 mM, red line: *β*-hydroxybutyrate 2 mM). The tripartite synapse system transitions from Regime D to Regime E below an average ATP generation rate of 1.17 × 10^6^ molecules ATP/second (dashed line). (b) There is a clear demarcation between Regime D and Regime E as a function of concentration of glucose. *β*HB = *β*-hydroxybutyrate.

It was previously shown that the tripartite synapse system functions in different regimes of operation depending on the availability of substrate for energy generation and neurotransmission [23]. We first evaluated the current model to determine the minimum ATP generation rate required for the tripartite synapse to fire in Regime D (vesicle-limited with superimposed ATP limitation), as opposed to the purely ATP-limited Regime E. This boundary region is of interest as Regime E could represent seizure-like activity at the level of the tripartite synapse [23]. We found that with ‘normal’ concentrations of *β*-hydroxybutyrate, the tripartite synapse system transitioned from Regime D into Regime E at a glucose concentration below 3.8 mM. With ‘elevated’ concentrations of *β*-hydroxybutyrate (2 mM), the transition occurred at a concentration below 3.1 mM glucose, demonstrating that *β*-hydroxybutyrate does indeed contribute significantly to ATP generation. The corresponding minimum average rate of ATP generation to maintain Regime D was 1.17 × 10^6^ molecules ATP/second (figure 3(a)). Thus, the rate of ATP generation can be used to distinguish between Regimes D and E.

**Figure 4.**
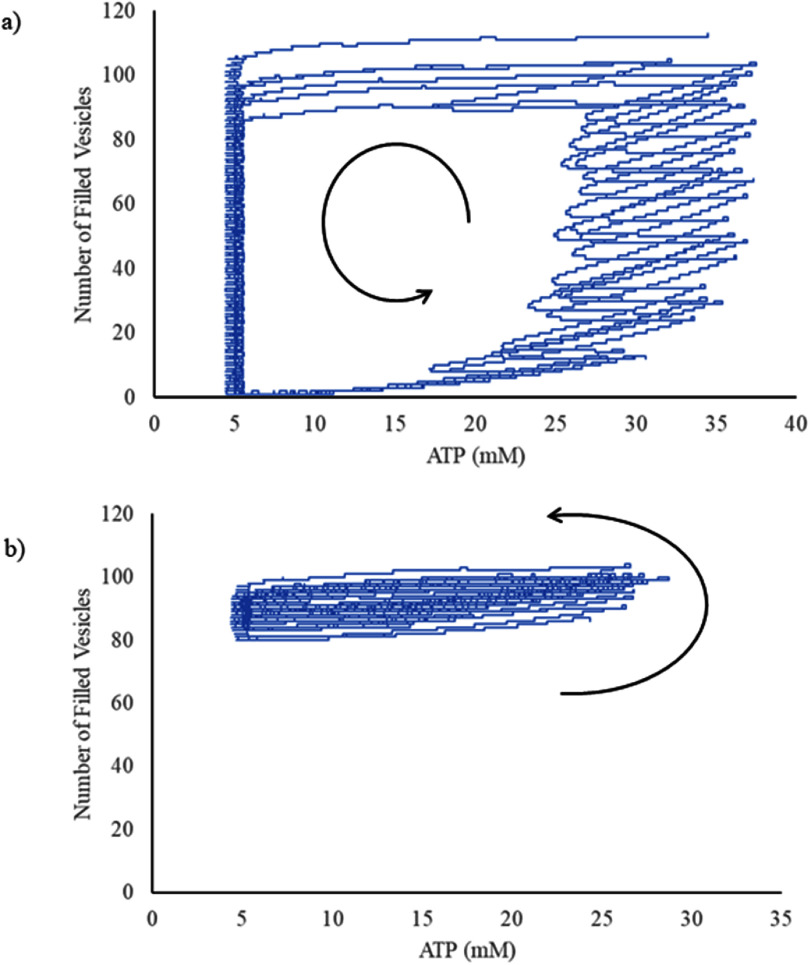
Limit cycles for Regime D and Regime E. Number of vesicles filled with glutamate is plotted against the concentration of ATP. The arrows represent the trajectory of the two species as a function of time. (a) Regime D. The limit cycle demonstrates the limitation caused by running out of filled vesicles: the number of filled vesicles oscillates between 1 and approximately 100. At the left side of the limit cycle, the concentration of ATP oscillates around 5 mM, and on the right, it increases to approximately 36 mM at maximum. (b) Regime E. The limit cycle has a very different appearance compared to that of Regime D. Although the concentration of ATP still oscillates around 5 mM at the left side of the limit cycle, the maximum ATP concentration only goes up to around 30 mM. More prominently, the number of filled vesicles oscillates between approximately 80 and 100, which is a significant departure from the limit cycle seen in Regime D.

We generated a phase plane diagram to investigate the bifurcation between Regime D and Regime E as a function of concentrations of glucose and *β*-hydroxybutyrate. We found that there was a crisp demarcation between the two regimes, which was a linear function of concentration of glucose, with an *R*^2^ goodness of fit of 0.9905 (figure 3(b)). We then plotted concentration of ATP versus number of vesicles filled with glutamate to demonstrate the limit cycles in Regime D and Regime E (figures 4(a) and (b)). The transition from Regime D to Regime E is a transition from one stable limit cycle to a second stable limit cycle with different characteristics.

### 3.2. Decreased availability of glucose leads to a state of rapid synaptic firing, which is rescued by addition of ketones

**Figure 5.**
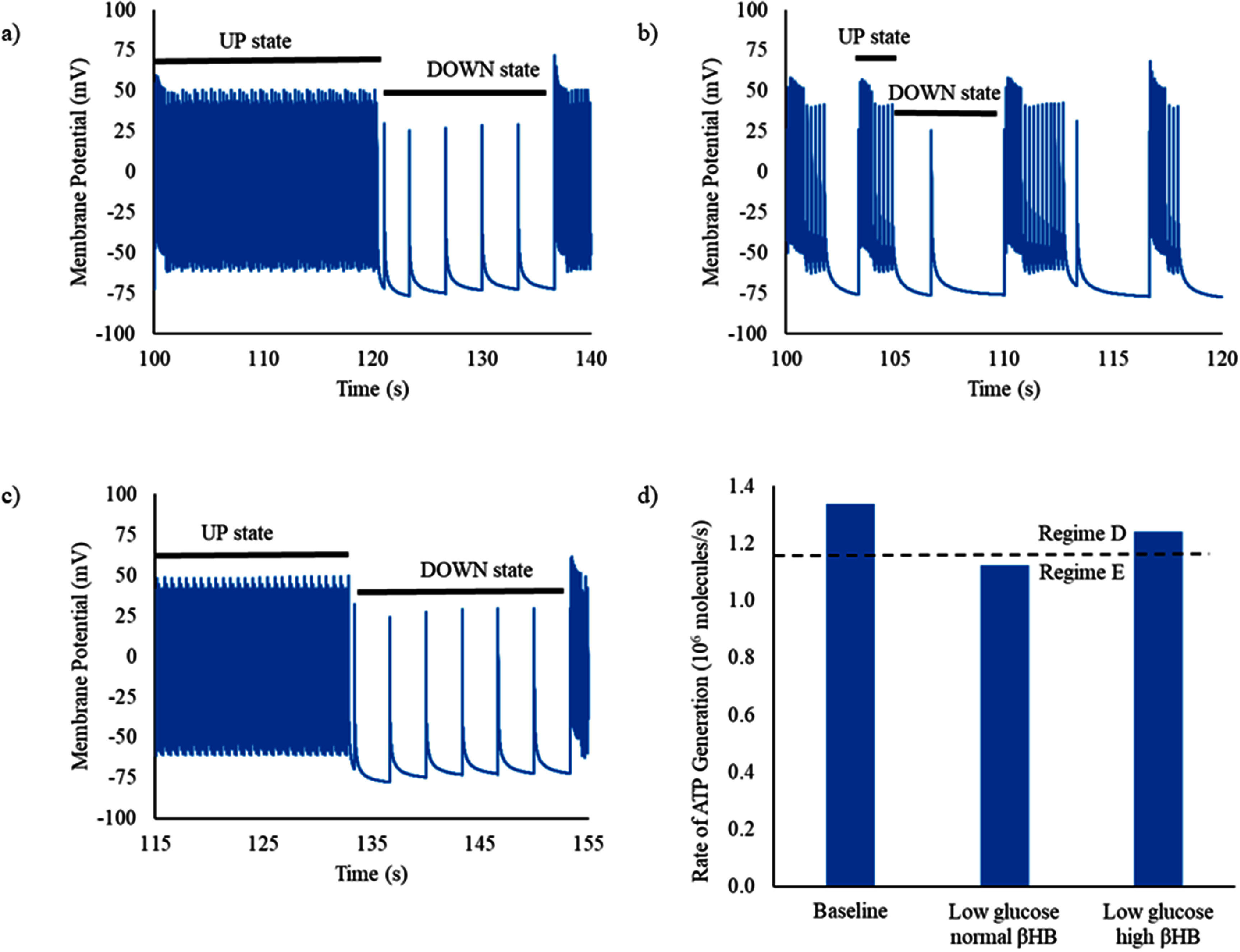
Rapid synaptic firing due to energy deficit is rescued with addition of ketone. (a) With normal glucose (5 mM = 90 mg dl^−1^) and normal *β*-hydroxybutyrate (0.25 mM) in the blood vessel, the tripartite synapse system functions in Regime D (vesicle-limited, with superimposed ATP limitation). Longer duration UP and DOWN states are present. (b) When the tripartite synapse has less glucose available (3.5 mM ≈ 63 mg dl^−1^), and *β*-hydroxybutyrate remains ‘normal’ (not homeostatically increased, at 0.25 mM), it transitions into Regime E. There are still UP states with rapid firing alternating with quiescent DOWN states, however both the UP and the DOWN states are significantly shorter in duration compared to in Regime D. (c) When the level of *β*-hydroxybutyrate is homeostatically increased as occurs in the ketogenic diet (2 mM), the tripartite synapse is able to return to Regime D. (d) At baseline glucose and ‘normal’ *β*-hydroxybutyrate, the average ATP generation rate in the presynaptic terminal is enough to maintain the tripartite synapse system in Regime D. With low glucose and ‘normal’ *β*-hydroxybutyrate, the average ATP generation rate in the presynaptic terminal drops below the minimum level required to sustain Regime D (dashed line). This is rescued by the increase in *β*-hydroxybutyrate. The average ATP generation rate is calculated from 100 s to 300 s to exclude the starting transient.

We simulated the behavior of the tripartite synapse in the conditions of normal concentrations of glucose and ketone, low glucose with normal ketone, and low glucose with elevated ketone (representing concentrations obtained with a ketogenic diet). At baseline (glucose 5 mM, *β*-hydroxybutyrate 0.25 mM), the tripartite synapse functions in Regime D—there are prolonged UP states with rapid firing and a relatively depolarized presynaptic resting membrane potential alternating with prolonged DOWN states with relatively infrequent firing and a relatively hyperpolarized resting membrane potential (figure 5(a)). In the condition of low glucose (3.5 mM ≈ 63 mg dl^−1^), but with ‘normal’ (not homeostatically elevated, 0.25 mM) *β*-hydroxybutyrate, the system goes into the ATP-limited Regime E, with brief bursts of rapid firing (figure 5(b)). When the level of *β*-hydroxybutyrate is increased to levels seen in the ketogenic diet (blood *β*-hydroxybutyrate 2 mM), the tripartite synapse is able to return to Regime D (figure 5(c)). The decrease in glucose concentration drops the average ATP generation rate in the presynaptic terminal to below the minimum level needed to maintain Regime D, and is rescued by the increase of *β*-hydroxybutyrate (figure 5(d)).

### 3.3. Addition of ketone rescues energy deficiency caused by decreased glucose transporter 1 protein activity

**Figure 6.**
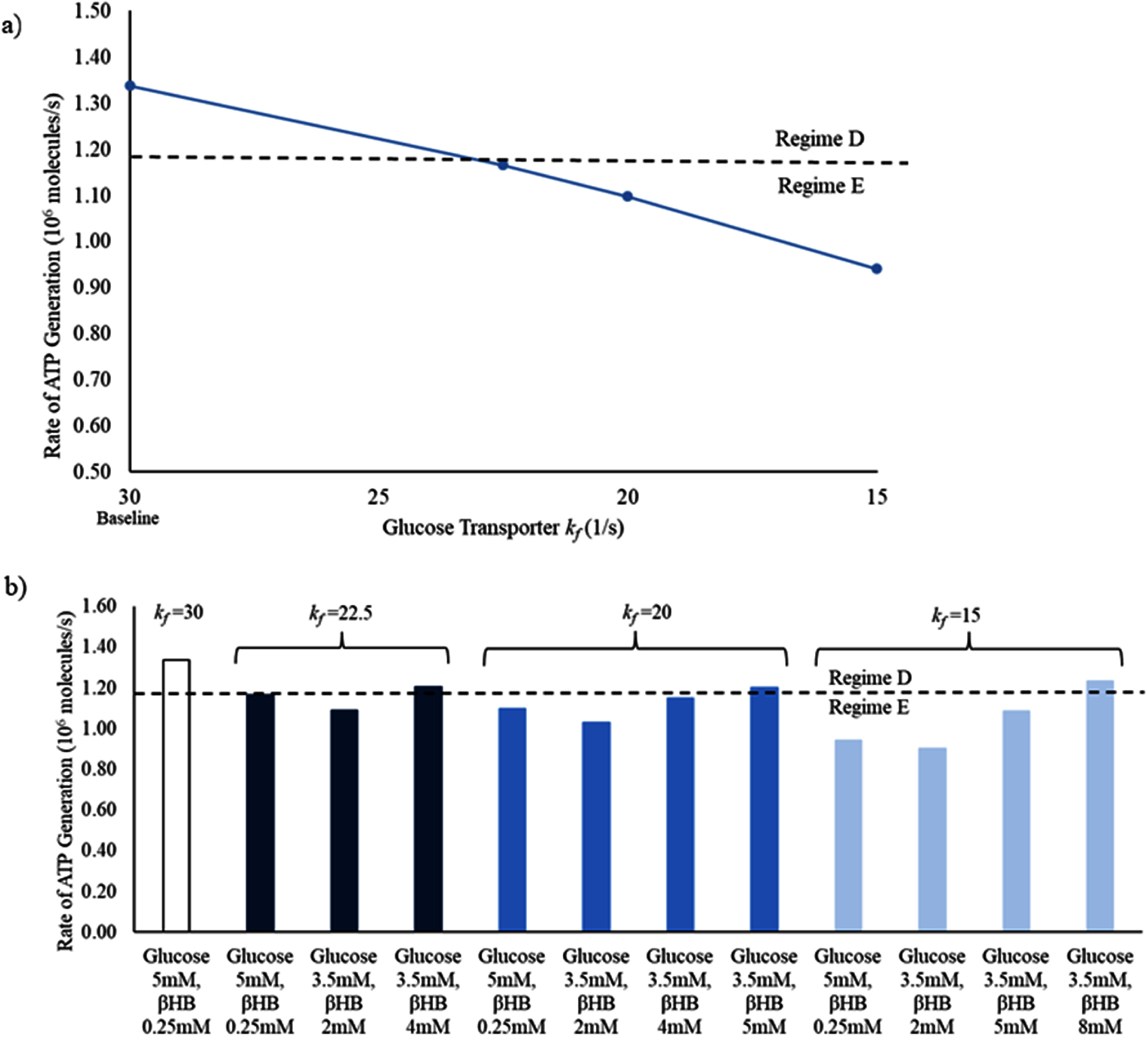
Rapid synaptic firing caused by deficiency of glucose transporter activity is rescued by ketones. (a) In the baseline condition (glucose 5 mM, *β*-hydroxybutyrate 0.25 mM), the tripartite synapse system functions in Regime D. When the GLUT1 transporter *k*_f_ is decreased, the average ATP generation rate in the presynaptic terminal decreases monotonically, and drops below the threshold to maintain Regime D (dashed line), and the tripartite synapse system functions in Regime E. (b) This is rescued in a dose-dependent manner by addition of increasing levels of *β*-hydroxybutyrate with a lower level of glucose (3.5 mM), as seen in the ketogenic diet. The lower the GLUT1 *k*_f_, the higher the concentration of *β*-hydroxybutyrate is required to return the tripartite synapse system to functioning in Regime D. The average rate of ATP generation was calculated from 100 s to 300 s to exclude the starting transient. *β*HB = *β*-hydroxybutyrate.

We next simulated the behavior of the tripartite synapse in several pathological conditions. The first condition investigated was the pathological decrease in the activity of the astrocytic glucose transporter (glucose transporter 1, GLUT1). This transporter is required for glucose to pass the blood-brain barrier to enter the central nervous system [33]. In the baseline state, with full activity of GLUT1, the tripartite synapse functions in Regime D. Decreasing the rate constant *k*_f_ of the glucose transporter led to a transition of the tripartite synapse system into Regime E due to decreased average ATP generation rate in the presynaptic terminal (figure 6(a)). This was rescued in the condition of the ketogenic diet (low glucose, high *β*-hydroxybutyrate) in a manner dependent on the level of *β*-hydroxybutyrate present—a lower GLUT1 *k*_f_ required higher levels of *β*-hydroxybutyrate for rescue (figure 6(b)).

### 3.4. Addition of ketone rescues energy deficiency caused by decreased activity of the pyruvate dehydrogenase complex

**Figure 7.**
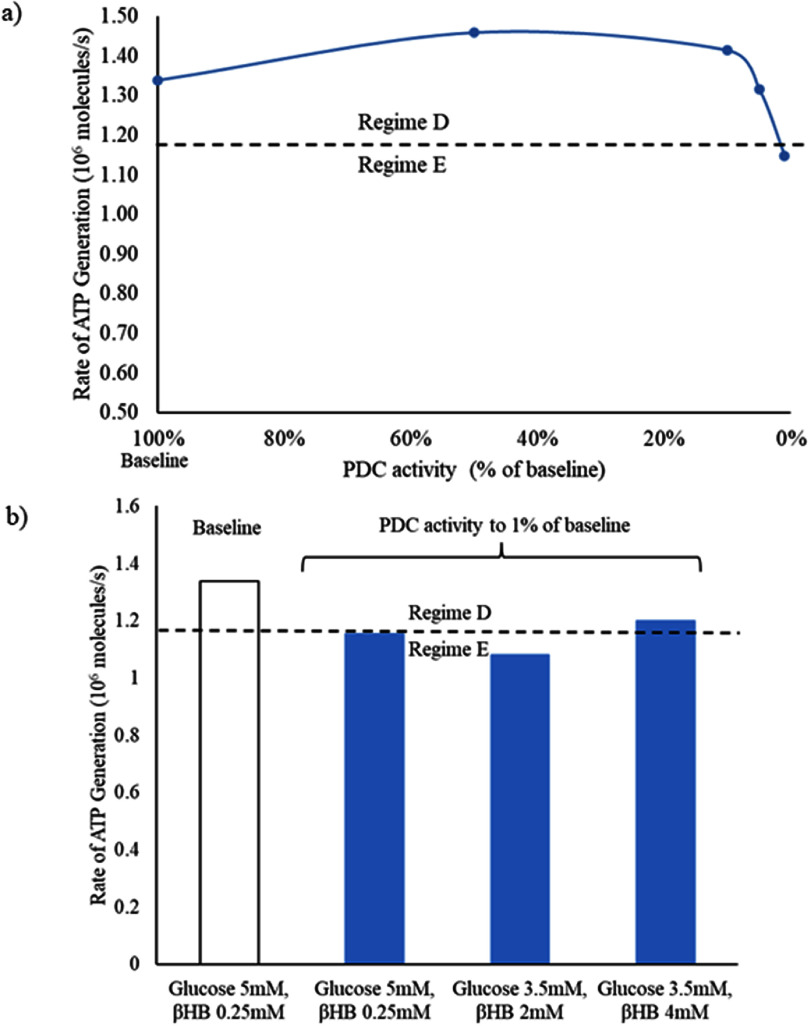
Rapid synaptic firing caused by deficiency of pyruvate dehydrogenase complex activity is rescued by ketones. (a) In the baseline state (glucose 5 mM and *β*-hydroxybutyrate 0.25 mM) with full activity of the pyruvate dehydrogenase complex, the tripartite synapse system functions in Regime D. When the rate constants for all of the reactions of the pyruvate dehydrogenase complex are decreased to 1% of baseline activity, the ATP generation rate drops below the minimum level required to maintain Regime D (dashed line). (b) This is rescued by addition of *β*-hydroxybutyrate (4 mM) with a lower level of glucose (3.5 mM), as is seen in the ketogenic diet. The system was not rescued by addition of 2 mM *β*-hydroxybutyrate. The average ATP generation rate was calculated from 100 s to 300 s to exclude the starting transient. PDC = pyruvate dehydrogenase complex. *β*HB = *β*-hydroxybutyrate.

We then evaluated the effects of the pathological decrease in the activity of the pyruvate dehydrogenase complex in the presynaptic terminal, astrocyte, and post-synaptic terminal. The pyruvate dehydrogenase complex is necessary for carbon substrate from pyruvate (from glycolysis) to enter into the tricarboxylic acid cycle as acetylCoA [34]. In the baseline state (glucose 5 mM, *β*-hydroxybutyrate 0.25 mM) with full activity of the pyruvate dehydrogenase complex, the tripartite synapse functions in Regime D. We then decreased the rates of all three reactions of the pyruvate dehydrogenase complex in each compartment (presynaptic terminal, post-synaptic terminal, and astrocyte). The reaction rate constants needed to be decreased to 1% of baseline activity in order for the average ATP generation rate in the presynaptic terminal to drop below the minimum ATP generation rate for Regime D (figure 7(a)). This was rescued in the condition of the ketogenic diet, with glucose 3.5 mM and *β*-hydroxybutyrate 4 mM; the system was not rescued by a *β*-hydroxybutyrate of 2 mM and stayed in Regime E for this concentration of ketone (figure 7(b)).

### 3.5. **Addition of ketone is unable to rescue energy shortage in** MCT**1 deficiency**

**Figure 8.**
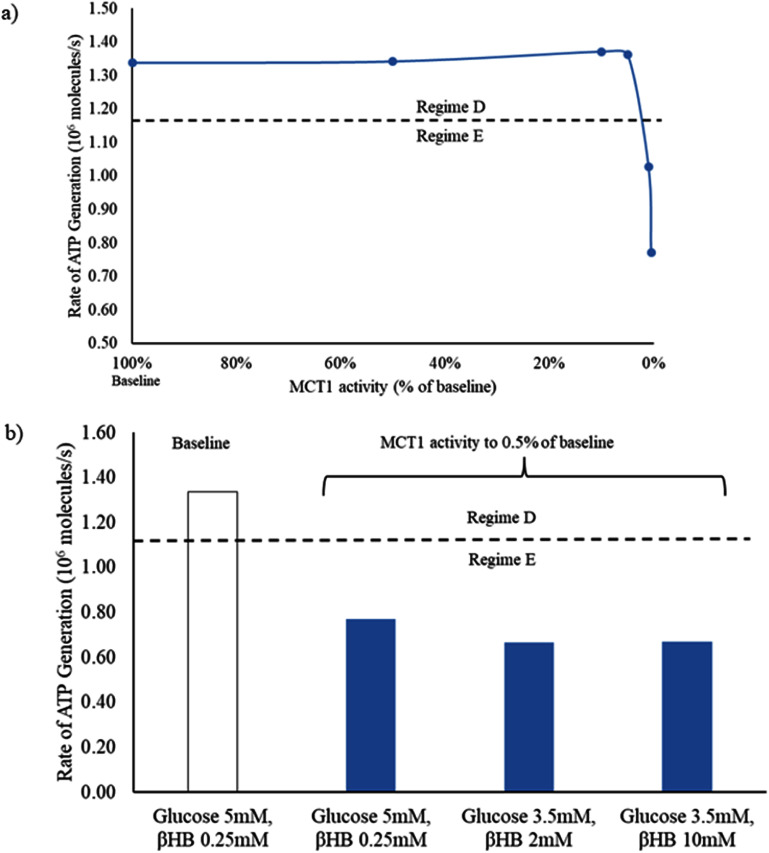
Rapid synaptic firing caused by deficiency of monocarboxylate transporter 1 activity cannot be rescued by the addition of ketones. (a) In the baseline state (glucose 5 mM, *β*-hydroxybutyrate 0.25 mM) with full monocarboxylate transporter 1 activity, the tripartite synapse system functions in Regime D. After the rate constant for the monocarboxylate transporter 1 was decreased to 0.5% of baseline activity, the ATP generation rate decreased below the minimum level required to maintain Regime D (dashed line), and the system transitioned to Regime E. (b) This could not be rescued even by significantly elevated levels of *β*-hydroxybutyrate (10 mM). The average ATP generation rate was calculated from 100 s to 300 s to exclude the starting transient. MCT1 = monocarboxylate transporter 1.

We investigated the effects of a pathological decrease in the activity of MCT1. This transporter transports multiple substrates including pyruvate, lactate, and *β*-hydroxybutyrate, with different affinities. It is prominently located on endothelial cells and on astrocytes; although neurons also have this transporter, it is normally expressed at much lower levels, if at all [35, 36]. In the baseline state (glucose 5 mM, *β*-hydroxybutyrate 0.25 mM) with full activity of MCT1, the tripartite synapse functions in Regime D. We decreased the reaction rate constants of transport for lactate and *β*-hydroxybutyrate in the blood vessel and astrocyte (but NOT the presynaptic or post-synaptic terminals). After the rate constants were decreased to 0.5% of baseline, the average ATP generation rate in the presynaptic terminal dropped below the minimum ATP generation rate required to maintain the system in Regime D (figure 8(a)). This was not rescued even with a concentration of *β*-hydroxybutyrate significantly above that typically seen in the ketogenic diet (glucose 3.5 mM, *β*-hydroxybutyrate 10 mM; figure 8(b)) [37, 38].

### 3.6. Mitochondrial complex I dysfunction cannot be rescued by addition of ketone

**Figure 9.**
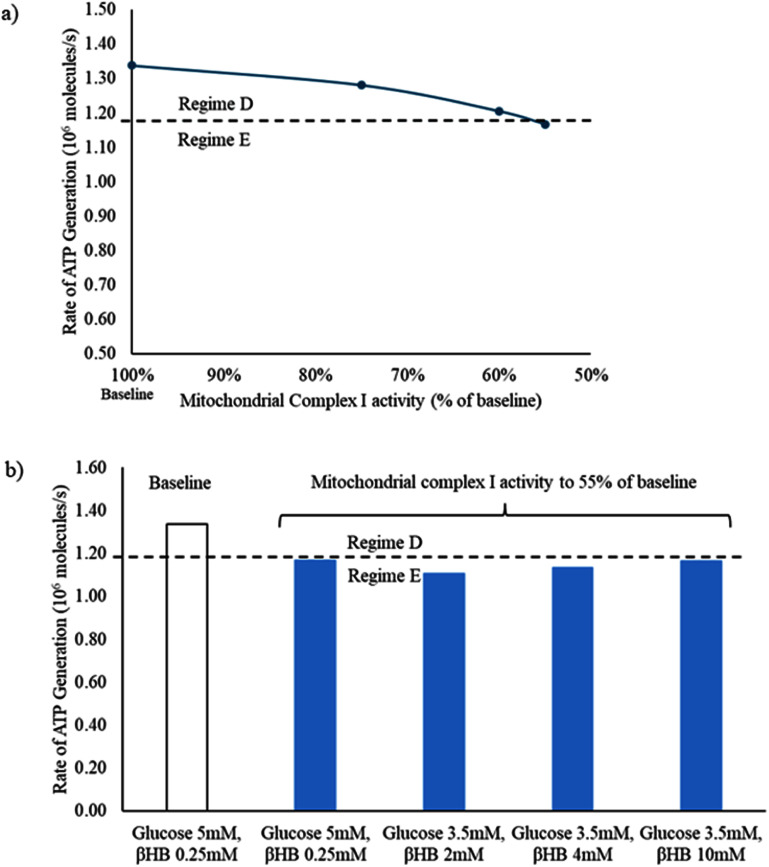
Rapid synaptic firing caused by deficiency of mitochondrial complex I activity cannot be rescued by the addition of ketones. (a) In the baseline state (glucose 5 mM, *β*-hydroxybutyrate 0.25 mM) with full activity of mitochondrial complex I, the tripartite synapse system functions in Regime D. After the reaction rate constants of mitochondrial complex I were decreased to 55% of baseline, the ATP generation rate decreased below the minimum level required to maintain Regime D (dashed line). (b) This could not be rescued even by very high levels of *β*-hydroxybutyrate (10 mM). The average ATP generation rate was calculated from 100 s to 300 s to exclude the starting transient. *β*HB = *β*-hydroxybutyrate.

Finally, we investigated the effects of a pathological decrease in the activity of mitochondrial complex I. This complex, NADH:CoQ oxidoreductase, is the largest enzyme of the electron transport chain, and is comprised of 44 different proteins [39]. It serves as the primary entry point for electrons into the electron transport chain, as it oxidizes NADH, one of the main products of the tricarboxylic acid cycle [34]. In the baseline state (glucose 5 mM, *β*-hydroxybutyrate 0.25 mM) with full activity of mitochondrial complex I, the tripartite synapse functions in Regime D. We decreased the reaction rate constants of complex I activity in the presynaptic terminal, post-synaptic terminal, and astrocyte. At 55% of the baseline reaction rate constants for mitochondrial complex I, the average ATP generation rate in the presynaptic terminal dropped below the minimum ATP generation rate required to maintain the system in Regime D (figure 9(a)). This could not be rescued even by very high levels of *β*-hydroxybutyrate (10 mM; figure 9(b)). A summary of all of the results is shown in table 2.

**Table 2.** Summary of simulations. *β*HB = *β*-hydroxybutyrate.

Experiment	Parameter	[*β*HB] at rescue	Outcome shown in:	Observation
Glucose dose response	Glucose = 2.6–5 mM	4–0 mM	Figure 3	Reduced glucose conditions are rescued in a dose-dependent manner by addition of *β*HB
Glucose concentration variation	Glucose = 5 mM Glucose = 3.5 mM	2 mM	Figure 5	Ketogenic diet can provide 3.5 mM glucose and 2 mM *β*HB in blood, which is able to rescue reduced glucose condition
Glucose transporter rate constant	*k*_f_ = 30 baseline *k*_f_ = 22.5 *k*_f_ = 20 *k*_f_ = 15	— 4 mM 5 mM 8 mM	Figure 6	Higher levels of *β*HB are required to rescue increased GLUT1 deficiency in a dose-dependent manner
Pyruvate dehydrogenase complex (pre- and post-synaptic terminals and astrocyte)	3 rate constants reduced from 100% activity down to 1% activity, in each domain	4 mM	Figure 7	*β*HB increase is able to provide acetylCoA as pyruvate pathway is diminished, and thus rescues its deficit.
MCT1 (astrocyte and blood vessels)	Rate constants reduced from 100% activity down to 0.5% activity, in each domain	Not rescued by 10 mM	Figure 8	MCT1 disruption affects both lactate and *β*HB pathways, and is thus difficult to rescue with *β*HB
Mitochondrial complex I (pre- and post-synaptic terminals and astrocyte)	Two rate constants reduced from 100% activity down to 55% activity, in each domain	Not rescued by 10 mM	Figure 9	Mitochondrial complex I is close to the last reaction in the production of ATP. *β*HB increase cannot rescue deficit at tested concentrations.

### 3.7. Certain key points in the energy pathway can significantly affect the mode of operation of the tripartite synapse

For the local sensitivity analysis, we ran a total of 47 * 11 * 2 = 1034 simulations. All of the parameters were kept constant except for the reaction rate constant that was being perturbed, and the rate constant(s) that represented the experimental condition. There were three primary patterns of sensitivity found (table 3). One pattern was that there was no change in the regime with perturbation of any of the reaction rate constants by 10%. A second pattern consisted of significant sensitivity to changes in parameters that affect upstream substrates, including glucose uptake; synthesis, export, or uptake of lactate; or uptake of *β*-hydroxybutyrate. The third pattern showed significant sensitivity to the specific reaction rate constant that was being varied in the experimental condition.

**Table 3.** Summary of results of sensitivity analysis. *β*HB = *β*-hydroxybutyrate.

Experiment	Blood concentrations	Mode	Parameter	Observation
Baseline	Glucose = 5 mM, *β*HB = 0.25 mM	Regime D	No sensitivity to ±10% variation	No mode shift observed
Reduction in blood glucose	Glucose = 3.5 mM, *β*HB = 0.25 mM	Regime E	*k*_f_, astrocytic glucose uptake from blood *k*_r_, astrocytic glucose uptake from blood *k*_f_, astrocytic pyruvate to lactate *k*_f_, astrocytic glucose to glucose-6-phosphate	Increased GLUT1, glycolysis step 1, and pyruvate to lactate all caused mode shift from Regime E to Regime D
Reduction in glucose with ketogenic diet	Glucose = 3.5 mM, *β*HB = 2 mM	Regime D	*k*_f_, astrocytic glucose uptake from blood *k*_r_, astrocytic glucose uptake from blood *k*_f_, astrocytic pyruvate to lactate *k*_f_, astrocytic lactate export to ECF *k*_f_, neuron lactate uptake *k*_f_, neuronal formation of succinylCoA	Variation in parameters that decrease glucose uptake, conversion of pyruvate into lactate in astrocyte and export and uptake of lactate from astrocyte and into the presynaptic terminal show significant sensitivity
50% Reduction in GLUT1 *k*_f_	Glucose = 5 mM, *β*HB = 0.25 mM	Regime E	No sensitivity to ±10% variation	No mode shift observed
50% Reduction in GLUT1 *k*_f_ with ketogenic diet	Glucose = 3.5 mM, *β*HB = 8 mM	Regime D	*k*_f_, *β*HB from blood vessel *k*_f_, neuronal oxidation of NADH by complex I *k*_f_, neuronal formation of succinylCoA *k*_f_, neuron lactate uptake *k*_f_, astrocytic lactate export to ECF *k*_f_, astrocytic pyruvate to lactate *k*_f_, astrocytic glucose uptake from blood *k*_r_, astrocytic glucose uptake from blood	Variation in parameters that decrease glucose uptake, conversion of pyruvate into lactate in astrocyte and export and uptake of lactate from astrocyte and into the presynaptic terminal show significant sensitivity
MCT1 reduced to 0.5% of baseline	Glucose = 5 mM, *β*HB = 0.25 mM	Regime E	No sensitivity to ±10% variation	No mode shift observed
MCT1 reduced to 0.5% of baseline with ketogenic diet	Glucose = 3.5 mM, *β*HB = 10 mM	Regime E	No sensitivity to ±10% variation	No mode shift observed
Mitochondrial complex I reduced to 55% of baseline	Glucose = 5 mM, *β*HB = 0.25 mM	Regime E	*k*_f_, neuronal oxidation of reduced complex I	Variation increasing value of the rate constant moves towards Regime D
Mitochondrial complex I reduced to 55% of baseline with ketogenic diet	Glucose = 3.5 mM, *β*HB = 10 mM	Regime E	*k*_f_, neuronal oxidation of reduced complex I *k*_f_, astrocytic lactate export to ECF	Variation increasing value of the rate constant moves towards Regime D
Pyruvate dehydrogenase complex reduced to 1% of baseline	Glucose = 5 mM, *β*HB = 0.25 mM	Regime E	*k*_f_, astrocytic glucose uptake from blood *k*_r_, astrocytic glucose uptake from blood *k*_f_, astrocytic pyruvate to lactate *k*_f_, astrocytic lactate export to ECF *k*_f_, astrocytic glucose to glucose-6-phosphate	Variation in parameters that increase glucose uptake, glycolysis step 1, conversion of pyruvate into lactate in astrocyte and export and uptake of lactate from astrocyte and into the presynaptic terminal show significant sensitivity
Pyruvate dehydrogenase complex reduced to 1% of baseline with ketogenic diet	Glucose = 3.5 mM, *β*HB = 4 mM	Regime D	*k*_f_, astrocytic glucose uptake from blood *k*_r_, astrocytic glucose uptake from blood *k*_f_, astrocytic pyruvate to lactate *k*_f_, astrocytic lactate export to ECF *k*_f_, *β*HB from blood vessel *k*_f_, astrocytic glucose to glucose-6-phosphate	Variation in parameters that decrease glucose uptake, glycolysis step 1, conversion of pyruvate into lactate in astrocyte and export and uptake of *β*HB from ECF and into the presynaptic terminal show significant sensitivity

To investigate the parameter ranges that led to state transitions, we performed 297 simulations with the baseline condition (glucose 5 mM, *β*-hydroxybutyrate 0.25 mM). We found that there were 5 forward reaction rate constants which led to a state transition of the system from Regime D to Regime E with a decrease in the value to 70%–80% of baseline. These included the forward reaction rate constant for the uptake of glucose into the astrocyte, the reaction rate constant for conversion of glucose to glucose-6-phosphate in the astrocyte, the reaction rate constant for conversion of glyceraldehyde-3-phosphate to 1,3-bisphosphoglycerate in the astrocyte, the forward reaction rate constant for conversion of pyruvate to lactate in the astrocyte, and the reaction rate constant for export of lactate from the astrocyte into the extracellular fluid. When the reverse reaction rate constant for uptake of glucose into the astrocyte was increased to 120%–130% of baseline, this also led to a state transition from Regime D to Regime E. The model was insensitive to changes in 30 parameters (no state change with a decrease in forward reaction rate constants to 1% of baseline or reverse rate constants to 100x of baseline). The model showed state transitions with intermediate changes from baseline for the remaining 11 parameters.

## 4. Discussion

In this work, we show that the addition of ketones in low-glucose conditions is capable of rescuing the function of the tripartite synapse, returning it from a pathological state of rapid firing (Regime E, which may represent a seizure-like equivalent at the synaptic level) to a more normal firing pattern (Regime D) [23]. The addition of different levels of ketone is also able to rescue synaptic function in a variety of other metabolic defects that impair the use of glucose and/or lactate. On the other hand, our model predicts that the introduction of ketones will not be able to normalize the function of the tripartite synapse for certain other metabolic conditions where the metabolic blockade cannot be bypassed by the ketone reactions. A sensitivity analysis revealed that the model was robust to most changes in kinetic reaction rate parameters in the energy pathway of the presynaptic terminal, although the model was expectedly sensitive to several parameters that significantly affected the availability of upstream substrate for the tricarboxylic acid cycle (glucose, lactate, and *β*-hydroxybutyrate). We propose that while the presence of ketones may have a variety of different effects, one of the major mechanisms of action is that they supply additional energy to the brain.

### 4.1. Energy deficiency as a final common pathway in seizures

Mitochondria are universally recognized for their important role in the production of ATP for use in cells [40–42]. Neurons are very metabolically active, and because their glycolytic capacity is limited, they are very dependent on mitochondrial oxidative phosphorylation for energy [43–45]. It is not surprising, then, that the nervous system is often affected in diseases of mitochondrial function, and that epilepsy is a frequent feature of mitochondrial diseases [40–42]. Although failure of energy production is a major hypothesis for the presence of epileptic seizures in patients with mitochondrial dysfunction, it should be noted that mitochondria also participate in other important neuronal functions including neurotransmitter synthesis, calcium homeostasis, cellular signaling, and cell death, which may also play a role in the development of epilepsy and seizures [41, 43]. Mitochondrial dysfunction in neurons may lead to a cascade of other effects leading to epileptogenesis, including increased excitability from hypofunction of the sodium-potassium ATPase, alterations of calcium sequestration, and/or enhanced neuronal and astrocytic glutamate release [40].

In addition to genetic mitochondrial diseases, an association has also been found between mitochondrial dysfunction and acquired epilepsies. It has been observed that during seizures, rates of glycolysis and lactate/pyruvate ratios are elevated, and between seizures, the same foci are hypometabolic [46]. Multiple lines of evidence have shown mitochondrial dysfunction in human patients with temporal lobe epilepsy and Ammon’s horn sclerosis [47]. In a more direct demonstration, it was found that there was deficiency of mitochondrial complex I in the epileptogenic tissue of patients who underwent epilepsy surgery [48]. Another group showed that there were significant alterations in levels of NAD(P)H in both a slice model and from chronic epileptic rats and humans, implicating mitochondrial dysfunction in these groups [49, 50]. In addition, increased production of reactive oxygen species (ROS) during epileptogenesis was shown to impair mitochondrial complex I function, potentially leading to a vicious cycle of increasing disruption of mitochondrial function and production of damaging oxidative species [51]. Further support for increased oxidative stress in epilepsy comes from the finding that the antioxidant couple of the reduced and oxidized forms of glutathione is shifted more towards the oxidized form in epileptic patients [52].

How might energy deficits in brain tissue lead to the generation of seizures? A common theory for seizure initiation is that excess excitation ‘overcomes’ inhibition, leading to seizure [53]. However, more recent evidence has suggested that it is in fact the GABAergic inhibitory neurons that may set off seizures [53, 54]. The mechanism of this shift has been thought to be due to paradoxical excitatory signaling due to excessive GABA release [55]. It has been found that interneurons are more susceptible to energy deficits, and that they may be running at close to their maximum metabolic ability in normal conditions [32]. Parvalbumin-containing fast-spiking interneurons reportedly have a particularly large amount of mitochondria, strongly implying that they require very high amounts of ATP to function [43]. Thus, disproportionate sensitivity to low energy conditions could in part explain the finding of loss of parvalbumin-positive GABAergic interneurons in sclerotic hippocampus of epilepsy patients [56]. In our model, we demonstrated abnormal seizure-like firing of an excitatory glutamatergic presynaptic terminal. It is expected that GABAergic interneuron presynaptic terminals will behave similarly in the context of energy deficits, and this may lead to the disordered firing that causes seizures at the level of the encephalogram (EEG).

### 4.2. Energy repletion as a primary mechanism of efficacy for the ketogenic diet

In this work, we showed that a threshold average rate of ATP generation was required in the presynaptic terminal in order for the tripartite synapse system to demonstrate normal UP and DOWN states (Regime D). When the average rate of ATP generation dropped below this threshold, the tripartite synapse system demonstrated a seizure-like firing pattern of brief bursts of rapid firing alternating with equally brief bursts of quiescence (Regime E). There was a clear bifurcation between Regime D and Regime E that depended on the concentrations of both glucose and *β*-hydroxybutyrate. In fact, the threshold is described as a simple linear function of the form a*[glucose] + b*[*β*-hydroxybutyrate] = *c* (where *a, b*, and *c* are positive constants), suggesting that higher levels of *β*-hydroxybutyrate can directly make up for lower levels of glucose in a dose-dependent manner. In addition, increased concentrations of *β*-hydroxybutyrate were able to directly ameliorate the energy deficit in several different pathological conditions: hypoglycemia, GLUT1 transporter deficiency, and pyruvate dehydrogenase complex deficiency. However, this replacement of substrate for energy production was only effective if the deficit occurred prior to the entry of acetylCoA into the tricarboxylic acid cycle: mitochondrial complex I deficiency could not be rescued even with high levels of *β*-hydroxybutyrate, because the deficit was too far downstream. Similarly, MCT1 deficiency could not be rescued by high levels of *β*-hydroxybutyrate, because the deficiency inhibited entry of *β*-hydroxybutyrate as well.

Deficiency of the GLUT1 glucose transporter leads to impaired transport of glucose into the central nervous system past the blood-brain barrier. This causes a well-described epileptic encephalopathy, which often demonstrates early-onset seizures, developmental delay, and movement disorders. In GLUT1 deficiency, there is a rapid and significant improvement in symptoms with institution of a ketogenic diet, and in many patients, other anti-seizure medications can be completely withdrawn [33]. Unsurprisingly, in our model, a decreased reaction rate (transport rate) of GLUT1 led to decreased glucose availability for the tripartite synapse, again leading to seizure-like firing in Regime E. As the glucose deficiency became more severe, more *β*-hydroxybutyrate was required to return the system to firing in Regime D. Interestingly, linear combinations of glucose and *β*-hydroxybutyrate concentrations sufficed to rescue the system. This is perhaps not surprising, as the catabolic pathways for glucose and *β*-hydroxybutyrate converge at acetylCoA, and the tripartite synapse is able to fire at a normal rate as long as it gets enough acetylCoA from any source. Just as seen in clinical practice, addition of *β*-hydroxybutyrate and reduction of glucose (mimicking the ketogenic diet), was able to rescue the function of the tripartite synapse.

Deficiency of the function of the pyruvate dehydrogenase complex, which converts pyruvate into acetylCoA for entry of carbon substrate into the tricarboxylic acid cycle, is another known mitochondrial metabolic defect. Although clinical manifestations in this deficiency are heterogenous, epilepsy is a prominent symptom of the deficiency. The ketogenic diet is a first line therapy for seizures due to pyruvate dehydrogenase complex deficiency in children [57, 58]. At a theoretical level, treatment with the ketogenic diet should at least partially rescue the phenotype in this disorder, as metabolism of *β*-hydroxybutyrate eventually leads to the production of acetylCoA, the deficient species. In our model, decreasing the activity of the pyruvate dehydrogenase complex led to manifestation of the seizure-like Regime E, and was again rescued by addition of sufficient amounts of *β*-hydroxybutyrate.

Deficiency of MCT1 is a rare genetic disorder in which patients with biallelic homozygous mutations have recurrent ketoacidosis, epilepsy, and developmental delay, while monoallelic carriers can have cyclic vomiting and ketoacidosis. Patients develop severe symptoms in ‘glucose shortage’ events such as conditions of fasting or infection, and are treated with intravenous glucose as well as sodium bicarbonate to correct the acidosis [59]. Individual cases have demonstrated other concurrent metabolic abnormalities, including lactic acidosis and hypoglycemia, during the periods of metabolic crisis [59, 60]. In our model, decreased activity of MCT1 again led to seizure-like firing of the tripartite synapse in Regime E. However, unlike deficiencies in the glucose transporter or pyruvate dehydrogenase complex, the institution of a high ketone, low glucose condition exacerbated the energy deficit rather than improving it. This finding was mirrored in a clinical case, where a patient with epilepsy and a heterozygous likely pathological mutation in MCT1 worsened clinically with institution of a ketogenic diet. Interestingly, her seizures decreased in frequency briefly, suggesting that neuronal hyperactivity decreased, although her metabolic status worsened [61]. MCT1 is involved in both the transport of lactate *and β*-hydroxybutyrate by the astrocyte, as well as transport of *β*-hydroxybutyrate into the ECF [35, 36]. As such, a defect in MCT1 would be expected to inhibit the transport of lactate into and out of the blood vessel compartment, the export of lactate from the astrocyte, the transport of *β*-hydroxybutyrate into the extracellular fluid, and the uptake of *β*-hydroxybutyrate into the astrocyte (though not the presynaptic or post-synaptic terminals). Because a ketogenic diet would theoretically lead to an alteration in the ratio of lactate to *β*-hydroxybutyrate, it is possible that instituting the diet could lead to mixed beneficial and adverse effects in MCT1 deficiency. This was in fact observed in our model as well, where the average ATP generation rate actually *increased* slightly with decreasing reaction rate constants for MCT1, prior to precipitously dropping. This was likely due to alteration of buffering of lactate within the blood vessel compartment, potentially with some contribution of metabolic compensation in the presynaptic terminal by utilizing more glutamate/*α*-ketoglutarate for energy.

Our model assumes the astrocyte neuronal lactate shuttle hypothesis, which means that one of the primary sources of energy for the presynaptic and post-synaptic terminals is lactate exported from the astrocyte [62]. This lactate export is decreased by the decreased activity of MCT1, decreasing the energy supply to the two neuronal compartments. In the context of the ketogenic diet, where ketone levels are high and glucose levels are low, astrocytes with MCT1 deficiency would suffer the dual blow of lower availability of glucose for glycolysis and inability to import ketone, leading to a more severe energy deficiency than would be present with a normoglycemic diet. This is the likely explanation for the worsening energy deficit in our model with high ketones and low glucose. The phenotype is not extremely severe however, as the neuronal terminals are still capable of oxidizing glutamate for energy. This suggests that MCT1 deficiency would be at least partially treated by increasing glucose supplies, which is in fact seen in case reports [59, 60].

As noted above, mitochondrial dysfunction frequently leads to manifestation of epilepsy. Mutations in mitochondrial complex I appear to be the most frequently associated with epilepsy, although other mitochondrial gene mutations have also been found [40, 63]. This is not surprising, as mitochondrial complex I is the largest enzyme complex in the electron transport chain, is the first complex in the electron transport chain, and is the entry point for electrons from the substrate (NADH) that ultimately leads to the production of a substantial fraction of the total ATP from respiration [34, 39]. Complex I comprises up to 40% of cases of respiratory chain disorders, and is the most frequently affected component of the electron transport chain [64]. The MTND5 gene, which encodes a subunit of mitochondrial complex I, appears to be a hotspot for mutations that lead to epilepsy [41]. We found that deficiency of mitochondrial complex I could not be rescued by addition of ketones. Interestingly, another group found that the ketogenic diet was effective for all of their studied patients with complex I deficiency, with five patients having >90% seizure reduction, and the remaining three patients having >50% reduction in seizure frequency [65].

One way to reconcile these findings is to note that the parameters in our model are static, and do not change homeostatically. Multiple studies have shown that the ketogenic diet produces profound changes in gene expression and mitochondrial function. The changes in gene expression expectedly include alterations in genes directly related to ketone metabolism such as the MCT1 and elements in the mitochondrial respiratory chain, increasing the ability to use ketones as energy substrate [36, 66]. In addition, it was previously shown that the ketogenic diet increases mitochondrial metabolism and flux through the tricarboxylic acid cycle, mitochondrial biogenesis, mitochondrial area, mitochondrial turnover, and mitochondrial respiration rate [13, 19, 67]. Increased enzyme translation and biogenesis of mitochondria would be expected to cause an effective increase in *all* of the mitochondrial enzymatic activities in our model, and this change would happen at a timescale of days to weeks (rather than the scale of minutes, in our model). Indeed, our sensitivity analysis showed that an increase in the mitochondrial complex I reaction rate constants by 10% (from 55% of baseline activity to 60.5% of baseline activity) was sufficient for the tripartite synapse system to return to Regime D, suggesting that a similar or greater increase of activity in patients would be sufficient to rescue their seizure phenotype. It has been shown that the number of mitochondrial profiles increased by more than 40% in animals that were fed a ketogenic diet, which is significantly higher than the increase required to rescue the tripartite synapse system in our model [19].

This then begs the question: why does all mitochondrial dysfunction not lead to epilepsy? It has been noted that (only) 20%–50% of patients with mitochondrial disease will have a seizure some time during the course of their disease, and (only) 35%–60% of pediatric patients with mitochondrial diseases have seizures ‘globally’ [42]. This means that up to approximately two-thirds of patients do not have seizures in spite of having mitochondrial disease. There are several possible explanations for this phenomenon. One is that it is well-known that mitochondria participate in functions beyond energy generation, including biosynthesis of important biochemical intermediates, cell signaling, calcium homeostasis, apoptosis, and autophagy [68–70]. As such, it is easy to imagine that there are mutations that affect some of these other functions without significantly affecting overall energy homeostasis. The picture is further muddied by the existence of 1) tissue-specific manifestations of disease, 2) ‘heteroplasmy’ (the presence of a high copy number of mitochondrial DNA, with different proportions of mutant and wild-type genes), 3) non-random segregation of mitochondrial DNA and 4) selective mitochondrial degradative pathways [68]. Finally, it has been noted that mitochondria function in different capacities in neurons versus astrocytes, between different types of neurons, and in different brain regions [41]. As such, there are many reasons why mitochondrial dysfunction may not manifest as an increased propensity for seizures, or that the mechanism of epilepsy may not be due to energy deficit. Nevertheless, given that a major function of mitochondria is energy generation, energy depletion is likely to be a major contributor to epileptogenesis and seizures.

### 4.3. Alternative or additional mechanisms of the ketogenic diet

It is also likely the case that the ketogenic diet has multiple diverse effects on the brain and body. Institution of the diet has been shown to modify gene expression of numerous other genes that are directly or indirectly related to energy metabolism, neurotransmission, and/or seizures. One group showed that there was a large-scale coordinated change in expression of transcripts related to synaptic transmission; this included both upregulation and downregulation of various ion channels, receptors, and transporters. The same group also showed primarily upregulation of the proteasome system [19]. Another group had previously shown that inhibition of the proteasomal system was correlated with decreased activity at mitochondrial complex I and complex II, increased generation of mitochondrial ROS, decreased mitochondrial turnover, and increased reliance on glycolysis for cellular energy requirements, suggesting that increasing the proteasomal system’s activity would reverse these effects [71]. Others have found that glutamate–cysteine ligase was upregulated for glutathione synthesis, which may help protect against injury caused by oxidative damage during seizures [14]. Alterations of the KCC2 chloride cotransporter were also found, which were thought to alter the effectiveness of the GABAergic system [72]. Effects on inflammation were also noted, with increased expression of the nuclear factor erythroid 2-related factor 2 (Nrf2) transcription factor, which can lead to protein expression that reduces oxidative stress and ROS [73]. In addition, the presence of ketones can inhibit activation of the NLRP3 inflammasome, thereby decreasing macrophage activation [74]. The ketogenic diet’s diverse, and not yet fully elucidated, effects may explain why it shows treatment efficacy in multiple different types of epilepsy and other neurological diseases.

Another player that has attracted more attention in the pathogenesis of epilepsy and seizures is the astrocyte. Astrocytes have been implicated in disruption of the blood-brain barrier, glial inflammation, disturbance of potassium clearance, impaired reuptake of neurotransmitters, and dysregulation of other neuromodulators. Because astrocytes create large intercellular networks using gap junctions, dysfunction of glial systems can affect the entire neuronal-glial network [75]. Interestingly, astrocytes are actually capable of synthesizing and exporting *β*-hydroxybutyrate themselves, although there is conflicting evidence regarding whether astrocyte-derived or liver-derived ketones are the primary source of the *β*-hydroxybutyrate in the brain [76, 77]. Ketones appear to have a profound impact on astrocytic function, including upregulation of the glutamate–glutamine cycle, which results in increased synthesis of glutamine and GABA, with decreased net synthesis of glutamate [77]. However, there is no clear evidence to suggest that astrocytic glutamate uptake is altered by presence of ketones. One group showed that although excitatory amino acid carrier 1 (EAAC1, also called excitatory amino acid transporter 3, EAAT3) mRNA was increased with the ketogenic diet, none of the protein levels of the glutamate transporters (EAAC1; GLT-1, glutamate transporter 1, also called EAAT2, excitatory amino acid transporter 2; or GLAST, glutamate aspartate transporter 1, also called EAAT1, excitatory amino acid transporter 1) were changed, nor was glutamate transport activity affected, at least in rats [78]. On the other hand, ketosis has been shown to both upregulate astrocytic aquaporin four and normalize astrocytic gap junction function, which are important for potassium homeostasis [77]. Thus, at least some of the beneficial effects of the ketogenic diet could be mediated by non-energy-related effects on astrocytes.

Inflammation has become another major area of interest in the field of epilepsy and seizures. Neuroinflammation that directly affects the neuronal-glial unit has been distinguished from systemic inflammation that primarily increases excitability through the loss of function of the blood-brain barrier [79]. Local neuroinflammation, as manifested by increased expression of inflammatory cytokines (such as interleukin 1*β*, IL-1*β* and tumor necrosis factor, TNF), danger signals (such as High Mobility Group Box 1, HMGB1), and associated receptors and ligands, has been found in resected foci of epileptogenic tissue. Other studies showed that inhibition of the IL-1 receptor (IL-1R)/Toll-like receptor 4 pathway decreased seizure frequency in both acute and chronic seizure models. Activation of these pathways was found to have diverse effects at neurons and astrocytes including increases of excitatory signaling/currents, decreases of inhibitory signaling/currents, elevated levels of extracellular glutamate, disruption of the blood-brain barrier, and disruption of astrocytic gap junctions [80]. Systemic inflammation and subsequent blood-brain-barrier dysfunction can also lead to increased excitability and seizures. A potential mechanism for this includes elevated extracellular potassium and dysregulated potassium homeostasis [79].

With the potential role for inflammation in seizures, much work has been done to elucidate potential anti-inflammatory effects of the ketogenic diet that can treat seizures. The diet has been found to inhibit NF-*κ*B, which then decreases the production of pro-inflammatory cytokines such as IL-1*β*, TNF-*α*, and IL-6. In addition, as noted above, the ketogenic diet induces an inhibition of the NLRP3 inflammasome, which again reduces the production of pro-inflammatory cytokines [81]. Interestingly, it was also found that there is an upregulation of the proinflammatory cytokine IL-17 and its receptor (IL-17R) in epileptogenic tissues, specifically expressed by pyramidal neurons, granule cells, reactive astrocytes, microglia, and endothelium [82]. Another group found that the number of IL-17-producing T cells in epileptogenic tissue positively correlated with the severity of seizures. In contrast, anti-inflammatory regulatory T cells (T-regs) were inversely correlated with disease severity [83]. The ketogenic diet has been shown to decrease levels of proinflammatory Th17 cells in the intestine, although it is not clear whether this effect extended to the brain [84]. Thus, the ketogenic diet may also beneficially affect inflammation in the brain, which may reduce the propensity for seizures.

A novel effect of the ketogenic diet on seizures may be related to modulation of circadian rhythms and sleep. It is well-known that transitions between sleep states can contribute to the onset of seizures, and that certain types of seizures have a predilection to certain stages of sleep [85]. Mechanistically, it has been proposed that the genes leading to circadian rhythms could either directly contribute to seizure susceptibility, or could influence neuronal excitability though downstream signaling pathways. Either pathway could alter the balance between excitatory and inhibitory neuronal activity, thereby leading to seizures [86]. Changes in both GABAergic and glutamatergic signaling pathways, as well as changes in astrocyte function, have been associated with circadian rhythms [87, 88]. In addition, various ion channels have also been found to be under clock-controlled expression [89]. As such, modulating sleep through effects on the circadian circuitry could also affect the propensity for seizures. Initiating a ketogenic diet has been found to alter circadian rhythms in various tissues including brain [90]. In addition, others have found that in normal subjects, there were trends towards reduced rapid eye movement (REM) sleep and increased slow wave sleep (SWS; also called non-REM sleep) with a ketogenic diet. On the other hand, in obese patients or those with epilepsy, sleep patterns normalized; in some cases, this consisted of increases in REM sleep and stable or decreased amounts of SWS. There were also improvements in sleep efficiency and sleep quality reported [91]. These changes in sleep quality and quantity could potentially correlate with normalization of the excitatory and inhibitory pathways under circadian influence, thereby contributing to reduction in seizures.

Finally, a brief word about intermittent fasting. Intermittent fasting encompasses a heterogenous group of feeding strategies wherein the time allotted to eating is limited, rather than the amount or composition of food. Intermittent fasting has been shown to have numerous effects on neuronal systems, including increasing the expression of brain-derived neurotrophic factor, peroxisome-proliferator-activated *γ* co-activator-1*α* (PGC-1*α*), sirtuin 3, and fibroblast growth factor 2. It also inhibits the mammalian target of rapamycin pathway, and activates autophagy [92]. It has been found that intermittent fasting can induce an anticonvulsant effect in models of seizures, and may also reduce astrogliosis [93]. Interestingly, however, a ketogenic diet and a caloric restriction diet (maintained by intermittent fasting) had differential ability to protect against seizures in several different seizure models, suggesting that ketogenic diets and caloric-restriction/intermittent fasting have non-overlapping anticonvulsant mechanisms from each other, at least in mice [94].

### 4.4. Why does the ketogenic diet not work for everyone?

It has been noted that the efficacy of the ketogenic diet is not related to the seizure type or frequency of seizures. These findings suggest that the mechanism(s) of the ketogenic diet are effective for epilepsy types beyond those known to be metabolic in origin. In addition, maximum efficacy is achieved days to weeks after the onset of the diet. Efficacy rapidly decreases, but does not completely revert to baseline, with a rapid reversal of ketosis [95]. In fact, expression of the MCT1 (but not MCT2 or MCT4) was reported to be significantly increased in mice fed a ketogenic diet, supporting the idea that the ketogenic diet induces changes in gene expression that facilitate use of ketones [36]. This implies that it is the ketones themselves that lead to efficacy of the ketogenic diet.

If energy deficit is a major cause for seizures, why do all patients not benefit from a ketogenic diet? One likely possibility is that not all patients achieve a therapeutic level of ketones for their specific epilepsy. It has been found that there was a correlation between serum levels of *β*-hydroxybutyrate and efficacy of the ketogenic diet for seizure reduction, and there is a suggestion that serum *β*-hydroxybutyrate levels need to be above approximately 4 mM for the ketogenic diet to be effective [37, 38]. In addition, another group found that there was an inverse relationship between the concentration of ketone bodies in the blood and the rate of glucose utilization. Interestingly, the relationship was not linear, as the authors observed that glucose utilization approached a minimum rate above a ketone concentration of approximately 5 mM [96]. It was also observed that some glucose oxidation was required for optimal usage of ketone bodies, and the authors of that work speculated that this may have been due to glucose facilitating the availability of (oxidized) nicotinamide adenine dinucleotide (NAD^+^) or supplying a basal amount of ATP [97]. Of note, red blood cells, which carry oxygen to various body tissues, are known to rely exclusively on glycolysis for ATP production, formation of 2,3-bisphosphoglycerate (which modulates oxygen delivery through its effect on hemoglobin), and generation of reduced nicotinamide adenine dinucleotide (NADH) [98]. We wonder whether impaired oxygen delivery due to hypoglycemia affecting red blood cells could explain the requirement for a basal level of glucose metabolism seen in the prior studies. This basal level of glucose metabolism would then limit the levels of ketone achievable by the ketogenic diet, to a level which may be below that needed by some individual patients to effectively treat their seizures. Even within metabolic-energetic causes for epilepsy, mutations downstream of the entry of acetylCoA would not be effectively treated by institution of the ketogenic diet. In this case, it may be that anaplerosis with other metabolic intermediates can rescue energy deficit and function more effectively than ketones alone [58].

There are also other possible reasons that a ketogenic diet may not work in a specific case for treatment of epilepsy. A long-standing hypothesis is that seizures are caused by an imbalance between excitation and inhibition [99]. In strong support of this hypothesis is the existence of numerous genetic ‘channelopathies’ that lead to epilepsy and seizures [100]. Dysfunction of ion channels has been implicated in acquired forms of epilepsy as well, both due to changes in plasticity after insult, as well as due to autoimmune attack [101]. Although it has been shown that ketones could affect expression of certain ion channels, it is very possible that these effects would not be sufficient to treat significant ion channel dysfunction [19]. It has been observed that although seizure-onset mechanisms are believed to be at the microscopic level, seizures themselves are a macroscopic (i.e. EEG-level) phenomenon [101–103]. As such, network connectivity is also important for seizure onset, and it is not clear that a ketogenic diet could address such aberrant connectivity. Furthermore, it has been found that numerous gene mutations, many of which are not directly related to ion channel function, or sometimes even synaptic transmission, can primarily or secondarily lead to epilepsy. These include mutations in genes that lead to structural brain abnormalities (and secondarily may cause epilepsy), genes that are involved in synaptic vesicle processing, genes mediating synaptic structural integrity, and genes contributing to the developmentally appropriate proliferation, migration, and differentiation of neurons and other brain cell types, among others [104–106]. Most of these abnormalities would be ‘baked’ into neuronal systems, making the underlying networks themselves seizure-prone. Finally, there is some evidence to suggest that after injury or with one of the above dysfunctions, there is a failure of neuronal homeostasis, which ultimately leads to the generation and maintenance of a seizure-prone network [99, 101]. The heterogenous nature of likely mechanisms of epilepsy certainly could contribute to the lower efficacy of the ketogenic diet.

### 4.5. Limitations

Our model does present some limitations, many of which are shared with the prior work [23]. A point of particular relevance is that in reduced realistic models such as this one, the kinetic parameters (such as reaction rate parameters) that would result in realistic model behavior cannot be expected to be the same as those that are measured in real systems. This is the case because the real systems also include numerous other reactions that are not included in the model, but nevertheless alter the dynamical behavior of the system. As an additional consequence, reduced realistic models will be able to show appropriate trends of behavior, but may not match exact thresholds for state changes in the system. For instance, although in our model, the activity of MCT1 had to be decreased by 99.5% from baseline in order for the system to show a state change (transition into Regime E), a real system may only require a decrease of 50% to show the analogous state change. It may be possible to arrive at reaction rate constants that yield state transitions close to those seen in real life, however because the parameter space is so large, finding that ideal combination of parameters remains a challenging problem.

Another limitation that has become more prominent in this model is the fact that the glucose supply is constant. Within the model, the concentration of glucose is held to be a boundary condition, which means that it remains constant no matter how rapidly the downstream reactions occur. Because the model includes first-order kinetics, this means that the rate of entry of glucose into the astrocyte always occurs at a constant rate. This is non-physiologic, and could alter the dynamics of depletion of various substrates. The same consideration applies to *β*-hydroxybutyrate.

### 4.6. Future directions

Our model predicts that provision of ketones can rescue function of energy-limited neurons and prevent seizure-like activity. This remains a computational study, and as such, we propose several potential experiments that could validate the findings. As previously noted, it was shown by another group that patients with mitochondrial complex I deficiency were actually benefited by institution of a ketogenic diet [65]. We speculated that this result may be due to increased mitochondrial biogenesis. One potential experiment to test this hypothesis would be to develop an *in vivo* animal model of mitochondrial complex I deficiency with seizures, which are rescued by administration of a ketogenic diet. If this improvement can be blocked or reversed in animals that have the PGC-1*α* pathway inhibited, a pathway known to regulate mitochondrial biogenesis, then this would suggest that mitochondrial biogenesis was the cause of improvement in the animals with mitochondrial complex I deficiency [107].

We found an unexpected sensitivity to perturbation of the reaction rate constant of the reaction that generates succinylCoA from *α*-ketoglutarate, in two experimental conditions (reduction of glucose with ketogenic diet, and reduction of GLUT1 transporter *k*_f_ with ketogenic diet). In reviewing reaction pathways, it is seen that succinylCoA is a necessary species in the mitochondrial conversion of acetoacetate into acetoacetylCoA. As such, it makes sense that a perturbation of the enzyme that generates succinylCoA could cause significant changes in the function of the tripartite synapse relying on ketones for generation of energy. This suggests that an alternative mechanism that produces additional succinylCoA could further benefit patients who have particularly low levels of glucose, and patients with glucose transporter deficiency who are being treated with the ketogenic diet. The branched chain amino acids isoleucine, valine, and methionine generate succinylCoA as part of their catabolism, with enzymes dependent on vitamin B12 and biotin [34]. Another potential set of experiments could be to generate an *in vivo* animal model with glucose transporter deficiency, or pharmacological blockade of the glucose transporter. The animals could then be treated with a level of ketone that does not rescue the seizure phenotype by itself, and then supplemented with vitamin B12, biotin, and branched chain amino acids, to see whether their phenotype can be rescued.

Finally, a question has been raised about the relative importance of ketogenesis, reduction of glycolysis, and anaplerosis in the efficacy of the ketogenic diet. One group recently found that in a fruit fly model of a severe mitochondrial encephalomyopathy (mutation of the subunit of mitochondrial complex V that allows passage of protons), the ketogenic diet produced a robust improvement in phenotype, as did the provision of an anaplerotic lipid, triheptanoin. Interestingly, the ketogenic diet was more effective than provision of saturating triheptanoin in the base mutation, however triheptanoin provided more benefit than the ketogenic diet in mutant flies in which isocitrate dehydrogenase (IDH, the rate-limiting enzyme of the tricarboxylic acid cycle) was also knocked down. This was thought to indicate that anaplerosis was less important than the presence of ketones themselves for efficacy of the ketogenic diet. However, anaplerosis from triheptanoin helped with the deficit of succinylCoA in the mutant flies with simultaneous knockdown of IDH [108]. Based on this finding, the importance of ATP generation as a mechanism of action for the ketogenic diet can be further tested. It was noted that the tricarboxylic acid cycle, in addition to providing reducing equivalents for the electron transport chain, also generates an ATP equivalent directly, through the action of the enzyme succinylCoA synthetase [34, 108]. This enzyme normally leads to formation of succinate from succinylCoA with the simultaneous formation of the ATP equivalent [34]. One *in vivo* experiment that can be performed is to create an animal model with a deficit in the electron transport chain (such as in complex V) leading to seizures, which is treated by the ketogenic diet. With blockade of the electron transport chain from the mutation, it would be expected that the primary sources of ATP are glycolysis (which is limited due to low availability of glucose in the ketogenic diet) and the reaction mediated by succinylCoA synthetase. In this setting, succinylCoA synthetase should be experimentally inhibited. If availability of ATP is a primary mechanism of efficacy of the ketogenic diet in this context, inhibition of succinylCoA synthetase should make the seizure phenotype worse. If not, the ketolytic reactions—specifically conversion of acetoacetate to acetoacetylCoA with simultaneous conversion of succinylCoA into succinate—should bypass the blockade of succinylCoA synthetase to provide succinate for the tricarboxylic acid cycle, and there should not be any significant effect. A summary of the proposed experiments can be found in table 4.

**Table 4.** Summary of proposed future experiments.

Area of investigation	Type	Description of experiment
Mitochondrial complex I deficiency	*In vivo*	1) In an animal model of seizures in mitochondrial complex I deficiency, demonstrate increased mitochondrial biogenesis and improvement of seizures with institution of the ketogenic diet. 2) Attempt to block the improvement in seizures by inhibiting the PGC-1*α* pathway, which regulates mitochondrial biogenesis.
Anaplerosis of succinylCoA	*In vivo*	1) In an animal model of glucose transporter deficiency (or pharmacological blockade of the glucose transporter), supplement with subtherapeutic levels of ketone and demonstrate incomplete rescue of seizure phenotype 2) Supplement with vitamin B12, biotin, and branched chain amino acids, and evaluate for improved rescue of the seizure phenotype.
Inhibition of succinylCoA synthetase	*In vivo*	1) In an animal model of mitochondrial electron transport chain deficiency (such as complex V deficiency) and seizures, demonstrate rescue with institution of the ketogenic diet. 2) In the animals ‘rescued’ by the ketogenic diet, inhibit succinylCoA synthetase, and evaluate for whether the seizure phenotype is worse.

## 5. Conclusions

We have demonstrated that addition of ketones is capable of rescuing dysfunction caused by energy deficit in a neurochemical model of the tripartite synapse. This occurs in conditions of low glucose supply, as well as in two well-known pathological conditions, glucose transporter 1 deficiency and pyruvate dehydrogenase complex deficiency, which lead to seizures treated with the ketogenic diet in the clinic. However, in our model, energy deficit is not rescued, and in fact worsens, in the pathological condition of MCT1 deficiency, a finding which is paralleled in a clinical case. Our model predicts that mitochondrial complex I deficiency should also not be rescued by addition of ketone, although anecdotal evidence has suggested benefit. This may be due to long-term changes in gene expression and enzyme activity that are not represented in our model. Our results indicate that the early hypothesis of the ketogenic diet replenishing depleted cellular energy stores is in fact a strong candidate for the primary mechanism of efficacy for the diet. We do not exclude other proposed mechanisms, and these would simply serve to improve the efficacy further. A greater appreciation of the contribution of energy deficits to seizure generation, and potentially even epileptogenesis, may lead to novel therapies in the future.

## Data Availability

The data that support the findings of this study are openly available at the following URL/DOI: https://doi.org/10.7910/DVN/6V9JWP [109].
